# Fish market prices drive overfishing of the ‘big ones’

**DOI:** 10.7717/peerj.638

**Published:** 2014-11-04

**Authors:** Athanassios C. Tsikliras, Konstantinos Polymeros

**Affiliations:** 1 Laboratory of Ichthyology, Department of Zoology, School of Biology, Aristotle University of Thessaloniki, Thessaloniki, Greece; 2 Department of Ichthyology and Aquatic Environment, University of Thessaly, Volos, Greece

**Keywords:** Overfishing, Body size, Market price, Trophic level, GAM

## Abstract

The relationship between fish market price and body size has not been explored much in fisheries science. Here, the mean market prices and fish body size were collected in order to examine the hypothesis that large fish, both among- and within-species, are being selectively targeted by fisheries because they may yield greater profit. Trophic levels, vulnerability to fishing and global landings were also collected because these variables may also be related to the market fish price. These relationships were examined using generalized additive models (GAM), which showed that, among species, fish market price was positively dependent on maximum total length (*P* = 0.0024) and negatively on landings (*P* = 0.0006), whereas it was independent of trophic level (*P* > 0.05) and vulnerability to fishing (*P* > 0.05). When the fish price vs. size relationship was tested within-species, large individuals were consistently attaining higher market prices compared to their medium and small-sized counterparts. We conclude that the selective removal of the larger fish, which is driven by their market price and to a lesser extent by their availability, may contribute to their overfishing.

## Introduction

Marine fish species account for over 80% of the total landings (Swartz, Sumaila & Watson, 2013) and a large proportion of them have been reported to be overexploited globally (e.g., Sumaila et al., 2012) and at various localities (e.g., Tsikliras, Tsiros & Stergiou, 2013). The overfishing trend is particularly true for large-bodied species such as sharks, rays, tunas, cod and halibut (Stevens et al., 2000; Field et al., 2009; Srinivasan et al., 2010), because for most species vulnerability to fishing increases with increasing body size (Anderson et al., 2008).

Fish market prices are among the economic drivers that affect fishers’ behaviour and, in particular, the selection of target species. Given that, according to economic theory, fishers make decisions based on potential profit (Brown, 2000), variations in price may make the fishing of a particular species more or less attractive (Holley & Marchal, 2004). Thus, fish species attaining higher market price (i.e., those with higher potential profit) are targeted first (Sethi, Branch & Watson, 2010). The direction and magnitude of the effects of market demand on biological populations and human behavior can depend on both biological attributes and institutional constraints (Reddy et al., 2013).

Apart from fish availability (the supply and demand relationship: Pinnegar et al., 2002), body size and catch weight may also determine fish price (Zimmermann, Heino & Steinshamn, 2011; Zimmermann, Steinshamn & Heino, 2011; Zimmermann & Heino, 2013). Size as one of several potential factors influencing the price of fish has been analyzed in multiple studies (e.g., Kristofersson & Rickertsen, 2004; Asche & Guillen, 2012). Although its effect has long been recognized (Gates, 1974; Gulland, 1982), the fish price and size relationship is rather overlooked in fisheries science, mainly due to the lack or inadequacy of data (Sumaila et al., 2007).

In the present work we examine the relationship of fish market price with body size, trophic level, abundance and vulnerability to fishing in fresh marine finfish and examine which of these variables determine the price of fish. The principal aim of the work was to test the hypothesis that large fish (the “big ones”), both among-species (i.e., large-bodied species) and within-species (i.e., large-sized individuals of a species), are being selectively targeted by fishers because they attain higher prices in the market and consequently may yield greater profit.

## Materials and Methods

In order to test the among-species hypothesis, we used the mean market prices (euros/kg) of 42 marine fish species for the period 1996–2010 by employing international trade database statistics recorded by Eurostat (http://epp.eurostat.ec.europa.eu/newxtweb), and collected the corresponding maximum reported total lengths (*L*_max_, cm), trophic levels (Troph) and vulnerability to fishing (Vuln), for each species, from FishBase (Froese & Pauly, 2012). We should point out, however, that maximum length may vary among stocks and does not necessarily correspond to the length structure of commercial catch, which also varies among stocks and depending on the fishing gear used.

In our analysis, we included only the price of fresh or chilled marine finfish (Code 0302 of Eurostat products) and excluded filleted or frozen products, which are being reported as separate records/codes. We also excluded entries that were grouped at higher taxonomic levels (genera, families and orders). Stationarity was not considered in the price time series because the fish price index is generally less volatile with fewer price spikes compared to other food price indices (Tveteras et al., 2012). The landings of each species (average values from 2000 to 2010) in European waters, as they appear in the FAO database (FAO, 2011), were used as an index of fish availability.

The effect of various predictor variables (i.e., maximum reported total length, trophic level, vulnerability and abundance index) on the price of fish (response variable) was explored by fitting a generalized additive model (GAM). GAMs were constructed in R software (R Development Core Team, 2008; version 2.15.2), using the ‘gam’ function of the ‘mgcv’ package (Wood, 2006). The predictor variables were smoothed using the cubic regression spline and the final model was selected using Akaike Information Criterion (AIC) value (Burnham & Anderson, 2002).

Since the among-species pattern may be the result of the scarcity (i.e., low supply with steady or increasing demand) of large-bodied species that boosts their prices (Pinnegar et al., 2002) and since the cost of fishing may be higher for larger species and may affect the fisher’s profit (Alder et al., 2008), we tested the same relationship within species, assuming that the fishing effort (hence the cost of fishing) and exploitation pattern remain more or less stable per fishing trip within a specific fishery (Seijo, Defeo & Salas, 1998).

In the within-species hypothesis, we downloaded the market prices (British pounds/kg) per size category of four commercial species of the northern Atlantic (Atlantic cod *Gadus morhua*, haddock *Melanogrammus aeglefinus*, plaice *Pleuronectes platessa*, and lemon sole *Microstomus kitt*). The prices per size category (small, medium, large; jumbo, i.e., very large, was a size category only for haddock) were taken from the market reports available in the FishUpdate magazine (www.fishupdate.com) for the period from March 2010 to September 2012, that were based on almost daily records of landings in two UK ports (Grimsby and Peterhead). From these -almost daily- landing records, a moving average value was calculated per size category and species. The moving average interval was set at 30 days. The mean market price values within species were compared with ANOVA and Tukey’s pairwise post-hoc test was used to compare the market price values per size category with each other.

## Results

The mean fish price ranged from 0.01 (for sprat *Sprattus sprattus*) to 9.17 euros/kg (for southern bluefin tuna *Thunnus maccoyii*), while the *L*_max_ ranged between 16 cm (sprat) and 470 cm (Atlantic halibut *Hippoglossus hippoglossus*). Trophic level ranged from 2.43 for pilchard *Sardinops* spp. to 4.53 for Atlantic halibut. Based on their exploitation status, halibut and tuna are, together with Atlantic cod, among the most heavily exploited marine fishes (Srinivasan et al., 2010), with very high vulnerability to fishing (Froese & Pauly, 2012).

**Figure 1:**
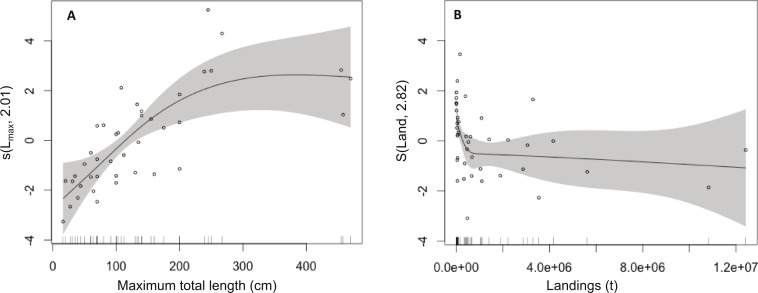
Effect of total length and landings on market price (among species). Modelled effects of (A) maximum total length (*L*_max_, cm) and (B) landings (Land, *t*) on fish market price. The solid line shows the fitted GAM function and the shaded areas indicate 95% confidence intervals. Relative density of data points (open circles) is indicated by the rug plot on the *x*-axis.

The GAM model results showed that there is a strong positive effect of *L_max_* (*P* = 0.0024) and a weaker positive effect of AI (*P* = 0.0006) on fish market price and that fish market price is independent of the trophic level and vulnerability to fishing (*P* > 0.05; Table 1, Fig. 1). These relationships clearly show that large-bodied species are sold at higher prices, compared to smaller species but their lower availability may also affect their market price (Fig. 1).

**Table 1:** Summary of the Gam model. Summary of the GAM model used to test the effect of various variables (*L*_max_, maximum total length, cm; Troph, trophic level; Vuln, vulnerability; Land, landings, *t*) on the market price of fish.

Variable	*df*	*F*	*P*	Significance	*n*	Deviance
*L*_max_	1.17	9.91	0.0024	**	42	77.2%
Troph	1.00	3.23	0.0795	ns		
Vuln	1.00	2.92	0.0989	ns		
Land	2.67	2.89	0.0006	***		
**Parametric coefficients**						
Intercept		3.120				
SE		0.179				
*t*		17.39				
*P*		<0.0010				
Adjusted *r*^2^		0.72				

**Figure 2:**
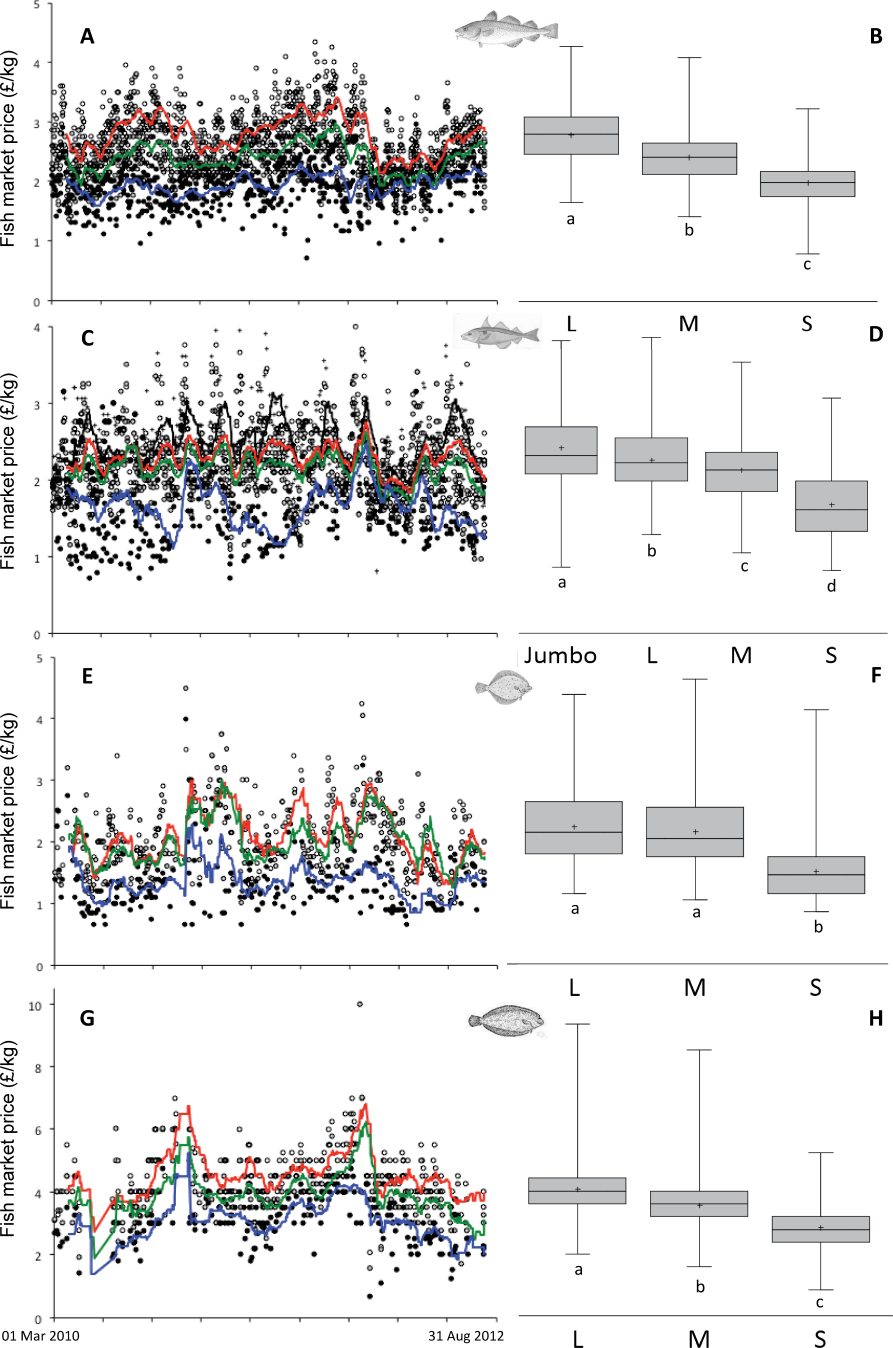
Within species variability in market price per size category. Moving average charts (open circles, red line: large; grey circles, green line: medium; black circles, blue line: small; crosses, black line: jumbo-used only for haddock) for Atlantic cod Gadus morhua (A), haddock *Melanogrammus aeglefinus* (C), plaice *Pleuronectes platessa* (E), and lemon sole *Microstomus kitt* (G) and box and whiskers plots (L: large, M: medium, S: small, J: jumbo used only for haddock) for Atlantic cod Gadus morhua (B), haddock *Melanogrammus aeglefinus* (D), plaice *Pleuronectes platessa* (F), and lemon sole *Microstomus kitt* (H). The rectangular part of the box and whiskers plot extends from the lower quartile to the upper quartile, covering the center half of each category. The center line within each box shows the location of the median and the plus sign indicates the location of the mean. The whiskers extend from the box to the minimum and maximum values. Mean values shown with the same letter within each plot are not significantly different from each other (*P* < 0.05).

Within-species, we found that large individuals (especially those of the Atlantic cod and lemon sole) were consistently attaining higher market prices compared to their medium and small-sized counterparts (ANOVA: *F* > 98, *P* < 0.001, in all cases; Fig. 2). The moving averages indicated that these trends remained constant throughout the study period (Figs. 2A, 2C, 2E, 2G). The mean price difference with body size was more pronounced for Atlantic cod (ANOVA: *F* = 732.3, *n* = 848, *P* < 0.001; Fig. 2B), haddock (ANOVA: *F* = 217.5, *n* = 806, *P* < 0.001; Fig. 2D) and lemon sole (ANOVA: *F* = 152.3, *n* = 224, *P* < 0.001; Fig. 2H) compared to plaice, for which there was not any statistical difference between large and medium size prices (ANOVA: *F* = 136, *n* = 267, *P* < 0.001; Fig. 2F), but the prices of small ones were lower from large/medium size prices. Thus, at least for the species studied in the present work and several other demersal and benthic ones, the large individuals of a species are consistently preferred by fishers because they are sold at higher prices, and, given that they are fished in the same trip with the cost of fishing remaining the same, are yielding higher profit. It is clear from Fig. 2 that the prices are subjected to seasonal fluctuations but the pattern remains the same (the larger fish are always sold at higher prices) and the periodicity is similar across size classes.

## Discussion

Fish species with potentially higher profit have been reported to be the first ones to be targeted (Sethi, Branch & Watson, 2010), and we show here that these are the bigger ones. Similar results have been recently reported for the Norwegian demersal and pelagic fisheries, where a significant positive relationship between weight and unit price in seven out of eight examined fish stocks was observed (Zimmermann & Heino, 2013). Within-species research has shown that larger individuals of several species in the Mediterranean Sea are sold at considerably higher prices (Colloca et al., 2013), although in some cases medium or “plate-sized’ fish are sold to restaurants at premium prices (Reddy et al., 2013). This could explain the saturation of price at large body sizes. The strong positive relationships between finfish market price and body size (within- and among-species) clearly indicate that market forces stimulate the targeting of larger fishes by fishing. Hence, as the “big ones” are removed at higher rates, their stock biomasses will be the first to decline. Evidence of gradual decline of large-bodied species stock biomass with time has been already reported (Pauly et al., 1998; Myers & Worm, 2003), although this down-fishing tend is questioned in several areas (Branch et al., 2010; but see also Stergiou & Tsikliras, 2011). The depletion of the “big ones” will, in turn, increase their prices because of their even lower supply and the vicious cycle continues. Given that the large-bodied species are more vulnerable to fishing because of their slow life history strategy (Jennings, Greenstreet & Reynolds, 1999), it is clear that their selective harvesting is unsustainable and disrupts ecosystem structure and function, i.e., leading to ecosystem overfishing (Murawski, 2000). Balanced harvesting (moderate fishing mortality across a wide range of species, stocks, and sizes) would mitigate adverse ecological effects of fishing and support sustainable fisheries by maintaining size and species composition (Garcia et al., 2012). In addition, size-dependent fish prices may change optimal fishing mortalities leading to unsustainable resource utilization (Zimmermann, Steinshamn & Heino, 2011).

Several years ago, the selective removal of larger and older individuals would have been a good fishing practice because it would relieve fishing pressure from the younger immature individuals and, thus, prevent growth overfishing (Beverton & Holt, 1957). However, the importance of the “big ones” to the reproductive potential of a stock has been recently recognized (Birkeland & Dayton, 2005) and current fisheries management will have to consider a slot of exploited lengths. In some cases, this might be beneficial because for some species, such as the Pacific red snapper (*Lutjanus peru*), medium or “plate-size” fish are sold at higher prices (Reddy et al., 2013) thereby protecting the small sized recruits and the large sized fecund individuals. The ecological and size selectivity through consumer and fisher behaviour, and fisheries management is one major cause for the current bad state of many marine ecosystems; hence there is a clear need of a paradigm shift (Mangel & Levin, 2005; Tudela & Short, 2005).

## Supplemental Information

10.7717/peerj.638/supp-1 Supplemental Information 1 List of species used in analysis
